# Stable isotope tracing in human plasma-like medium reveals metabolic and immune modulation of the glioblastoma microenvironment

**DOI:** 10.1093/neuonc/noaf248

**Published:** 2025-10-25

**Authors:** Milan R Savani, Mohamad El Shami, Kenji Miki, Lauren C Gattie, Bailey C Smith, William H Hicks, Jacob O Weiss, Skyler S Oken, Lavanya N Katta, Tracey Shipman, Maged T Ghoche, Lauren G Zacharias, Misty S Martin-Sandoval, Eric Y Montgomery, Yi Xiao, Diana D Shi, Jeremy N Rich, Timothy E Richardson, Pascal O Zinn, Bradley C Lega, Thomas P Mathews, Ralph J DeBerardinis, Kalil G Abdullah, Samuel K McBrayer

**Affiliations:** Children’s Medical Center Research Institute, UT Southwestern Medical Center, Dallas (M.R.S., B.C.S., W.H.H., J.O.W., L.N.K., T.S., L.G.Z., M.S.M.-S., E.Y.M., Y.X., D.D.S., T.P.M., R.J.D., S.K.M.); Department of Neurosurgery, University of Pittsburgh School of Medicine, Pittsburgh (M.E.S., K.M., L.C.G., W.H.H., S.S.O., M.T.G., P.O.Z., K.G.A.); Hillman Comprehensive Cancer Center, University of Pittsburgh Medical Center, Pittsburgh (M.E.S., K.M., L.C.G., W.H.H., S.S.O., M.T.G., K.G.A.); Department of Neurosurgery, University of Pittsburgh School of Medicine, Pittsburgh (M.E.S., K.M., L.C.G., W.H.H., S.S.O., M.T.G., P.O.Z., K.G.A.); Hillman Comprehensive Cancer Center, University of Pittsburgh Medical Center, Pittsburgh (M.E.S., K.M., L.C.G., W.H.H., S.S.O., M.T.G., K.G.A.); Department of Neurosurgery, University of Pittsburgh School of Medicine, Pittsburgh (M.E.S., K.M., L.C.G., W.H.H., S.S.O., M.T.G., P.O.Z., K.G.A.); Hillman Comprehensive Cancer Center, University of Pittsburgh Medical Center, Pittsburgh (M.E.S., K.M., L.C.G., W.H.H., S.S.O., M.T.G., K.G.A.); Children’s Medical Center Research Institute, UT Southwestern Medical Center, Dallas (M.R.S., B.C.S., W.H.H., J.O.W., L.N.K., T.S., L.G.Z., M.S.M.-S., E.Y.M., Y.X., D.D.S., T.P.M., R.J.D., S.K.M.); Children’s Medical Center Research Institute, UT Southwestern Medical Center, Dallas (M.R.S., B.C.S., W.H.H., J.O.W., L.N.K., T.S., L.G.Z., M.S.M.-S., E.Y.M., Y.X., D.D.S., T.P.M., R.J.D., S.K.M.); Department of Neurosurgery, University of Pittsburgh School of Medicine, Pittsburgh (M.E.S., K.M., L.C.G., W.H.H., S.S.O., M.T.G., P.O.Z., K.G.A.); Hillman Comprehensive Cancer Center, University of Pittsburgh Medical Center, Pittsburgh (M.E.S., K.M., L.C.G., W.H.H., S.S.O., M.T.G., K.G.A.); Children’s Medical Center Research Institute, UT Southwestern Medical Center, Dallas (M.R.S., B.C.S., W.H.H., J.O.W., L.N.K., T.S., L.G.Z., M.S.M.-S., E.Y.M., Y.X., D.D.S., T.P.M., R.J.D., S.K.M.); Department of Neurosurgery, University of Pittsburgh School of Medicine, Pittsburgh (M.E.S., K.M., L.C.G., W.H.H., S.S.O., M.T.G., P.O.Z., K.G.A.); Hillman Comprehensive Cancer Center, University of Pittsburgh Medical Center, Pittsburgh (M.E.S., K.M., L.C.G., W.H.H., S.S.O., M.T.G., K.G.A.); Children’s Medical Center Research Institute, UT Southwestern Medical Center, Dallas (M.R.S., B.C.S., W.H.H., J.O.W., L.N.K., T.S., L.G.Z., M.S.M.-S., E.Y.M., Y.X., D.D.S., T.P.M., R.J.D., S.K.M.); Children’s Medical Center Research Institute, UT Southwestern Medical Center, Dallas (M.R.S., B.C.S., W.H.H., J.O.W., L.N.K., T.S., L.G.Z., M.S.M.-S., E.Y.M., Y.X., D.D.S., T.P.M., R.J.D., S.K.M.); Department of Neurosurgery, University of Pittsburgh School of Medicine, Pittsburgh (M.E.S., K.M., L.C.G., W.H.H., S.S.O., M.T.G., P.O.Z., K.G.A.); Hillman Comprehensive Cancer Center, University of Pittsburgh Medical Center, Pittsburgh (M.E.S., K.M., L.C.G., W.H.H., S.S.O., M.T.G., K.G.A.); Children’s Medical Center Research Institute, UT Southwestern Medical Center, Dallas (M.R.S., B.C.S., W.H.H., J.O.W., L.N.K., T.S., L.G.Z., M.S.M.-S., E.Y.M., Y.X., D.D.S., T.P.M., R.J.D., S.K.M.); Children’s Medical Center Research Institute, UT Southwestern Medical Center, Dallas (M.R.S., B.C.S., W.H.H., J.O.W., L.N.K., T.S., L.G.Z., M.S.M.-S., E.Y.M., Y.X., D.D.S., T.P.M., R.J.D., S.K.M.); Children’s Medical Center Research Institute, UT Southwestern Medical Center, Dallas (M.R.S., B.C.S., W.H.H., J.O.W., L.N.K., T.S., L.G.Z., M.S.M.-S., E.Y.M., Y.X., D.D.S., T.P.M., R.J.D., S.K.M.); Children’s Medical Center Research Institute, UT Southwestern Medical Center, Dallas (M.R.S., B.C.S., W.H.H., J.O.W., L.N.K., T.S., L.G.Z., M.S.M.-S., E.Y.M., Y.X., D.D.S., T.P.M., R.J.D., S.K.M.); Children’s Medical Center Research Institute, UT Southwestern Medical Center, Dallas (M.R.S., B.C.S., W.H.H., J.O.W., L.N.K., T.S., L.G.Z., M.S.M.-S., E.Y.M., Y.X., D.D.S., T.P.M., R.J.D., S.K.M.); Department of Radiation Oncology, Dana-Farber/Brigham and Women’s Cancer Center, Harvard Medical School, Boston (D.D.S.); Lineberger Comprehensive Cancer Center, University of North Carolina at Chapel Hill, Chapel Hill (J.N.R.); Department of Neurology, University of North Carolina at Chapel Hill, Chapel Hill (J.N.R.); Department of Pathology, Molecular and Cell-Based Medicine, Icahn School of Medicine at Mount Sinai, New York (T.E.R.); Department of Neurosurgery, University of Pittsburgh School of Medicine, Pittsburgh (M.E.S., K.M., L.C.G., W.H.H., S.S.O., M.T.G., P.O.Z., K.G.A.); Department of Neurosurgery, UT Southwestern Medical Center, Dallas (B.C.L.); Children’s Medical Center Research Institute, UT Southwestern Medical Center, Dallas (M.R.S., B.C.S., W.H.H., J.O.W., L.N.K., T.S., L.G.Z., M.S.M.-S., E.Y.M., Y.X., D.D.S., T.P.M., R.J.D., S.K.M.); Children’s Medical Center Research Institute, UT Southwestern Medical Center, Dallas (M.R.S., B.C.S., W.H.H., J.O.W., L.N.K., T.S., L.G.Z., M.S.M.-S., E.Y.M., Y.X., D.D.S., T.P.M., R.J.D., S.K.M.); Harold C. Simmons Comprehensive Cancer Center, UT Southwestern Medical Center, Dallas (R.J.D., S.K.M.); Eugene McDermott Center for Human Growth and Development, UT Southwestern Medical Center, Dallas (R.J.D.); Howard Hughes Medical Institute, UT Southwestern Medical Center, Dallas (R.J.D); Department of Neurosurgery, University of Pittsburgh School of Medicine, Pittsburgh (M.E.S., K.M., L.C.G., W.H.H., S.S.O., M.T.G., P.O.Z., K.G.A.); Hillman Comprehensive Cancer Center, University of Pittsburgh Medical Center, Pittsburgh (M.E.S., K.M., L.C.G., W.H.H., S.S.O., M.T.G., K.G.A.); Children’s Medical Center Research Institute, UT Southwestern Medical Center, Dallas (M.R.S., B.C.S., W.H.H., J.O.W., L.N.K., T.S., L.G.Z., M.S.M.-S., E.Y.M., Y.X., D.D.S., T.P.M., R.J.D., S.K.M.); Harold C. Simmons Comprehensive Cancer Center, UT Southwestern Medical Center, Dallas (R.J.D., S.K.M.); Peter O’Donnell Jr. Brain Institute, UT Southwestern Medical Center, Dallas (S.K.M.)

**Keywords:** glioma, metabolism, organoids, preclinical models, stable isotope tracing

## Abstract

**Background:**

In vivo stable isotope tracing is useful for natively surveying glioma metabolism but can be difficult to implement. Stable isotope tracing is tractable using in vitro glioma models, but most models lack nutrient conditions and cell populations relevant to human gliomas. This limits our ability to study glioma metabolism in the presence of an intact tumor microenvironment (TME) and immune-metabolic crosstalk.

**Methods:**

We optimized an in vitro stable isotope tracing approach for human glioma explants and glioma stem-like cell (GSC) lines that integrates human plasma-like medium (HPLM). We performed ^15^N_2_-glutamine tracing in GSC monocultures and human IDH-wildtype glioblastoma explants and developed an analytical framework to evaluate microenvironment-dependent metabolic features that distinguish them. We also conducted spatial transcriptomics to assess transcriptional correlates to metabolic activities.

**Results:**

Human plasma-like medium culture preserved glioma explant viability and stemness while unmasking metabolic and immune programs suppressed by conventional culture conditions. Stable isotope tracing in HPLM revealed TME-dependent and TME-independent features of tumor metabolism. Tissue explants recapitulated tumor cell-intrinsic metabolic activities, such as synthesis of immunomodulatory purines. Unlike GSC monocultures, tissue explants captured tumor cell-extrinsic activities associated with stromal cell metabolism, as exemplified by astrocytic guanosine diphosphate mannose production in heterocellular explants. Finally, glioma explants displayed tumor subtype-specific metabolic reprogramming, including robust pyrimidine degradation in mesenchymal cells.

**Conclusions:**

We present a tractable approach to assess glioma metabolism in vitro under physiologic nutrient levels and in the presence of an intact TME. This platform opens new avenues to interrogate glioma metabolism and its interplay with the immune microenvironment.

## Graphical Abstract

**Graphical Abstract:**
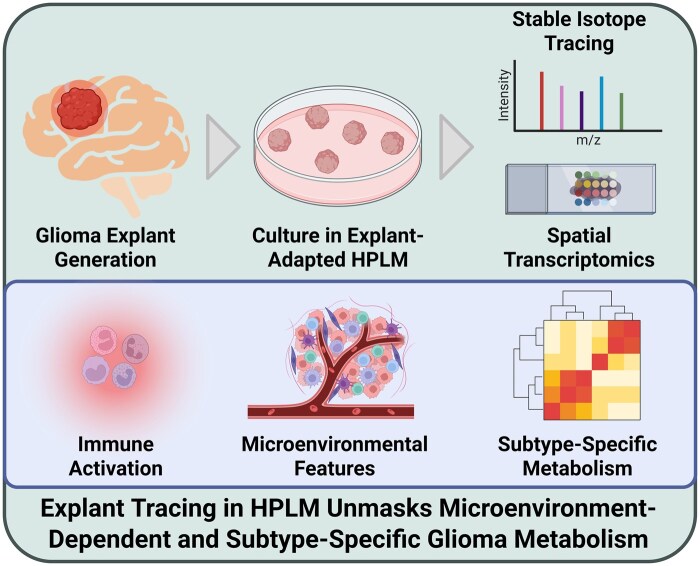

Key Points

- Human plasma-like medium supports culture of glioma explants and stimulates metabolic and immune transcriptional responses.
- Stable isotope tracing in glioma explants reveals contributions of tumor cells, stromal cells, and gene expression programs to tumor metabolism.

Importance of the Study

Metabolic reprogramming is a hallmark of tumor biology, but in vitro studies of glioma metabolism often fail to replicate the nutrient complexity and cellular heterogeneity of the tumor microenvironment (TME). We developed a method to perform stable isotope tracing in glioma explants grown in human plasma-like medium (HPLM) to analyze metabolism in a nutrient context that reflects in vivo conditions. By comparing metabolic activities between glioma cell monocultures and explanted tumor tissues, our approach captures features of tumor metabolism that are driven by the microenvironment. We show that HPLM not only sustains cell fitness and identity in tumor explants but also evokes distinct metabolic patterns and immune activation signatures repressed by standard culture conditions. Our approach offers a tractable and scalable way to study tumor cell intrinsic and microenvironmental metabolism in faithful tissue culture glioma models, complementing powerful yet low-throughput in vivo stable isotope tracing approaches.

Metabolic reprogramming is a hallmark of cancer biology that provides energy and substrates for cell growth.^1^^,^^2^ There has been substantial interest in the metabolic adaptations that enable glioma cells to grow and proliferate, which has resulted in the nomination of new therapeutic targets.^3–17^ Tumor-relevant biochemical networks are flexible and sensitive to concentrations of environmental nutrients and the metabolic activities of neighboring cells.^18^^,^^19^ The gold standard for investigating glioma metabolism while capturing microenvironmental interactions is to utilize in vivo stable isotope tracing in mouse brain tumor models or in patients.^20–26^ These experiments are technically challenging, costly, and time-consuming. However, performing stable isotope tracing in more accessible systems, such as cell culture models, may not capture vital characteristics of tumor metabolism, including heterocellular interactions of the tumor microenvironment (TME) and physiologic nutrient levels.

Culture media that more accurately reflect physiologic nutrient availability—such as human plasma-like medium (HPLM)—have recently been applied to cancer models and reveal marked effects on cellular metabolism and nutrient utilization.^3^^,^^27–29^ Human plasma-like medium contains human-relevant concentrations of typical media components such as glucose, amino acids, and salt ions, as well as some metabolites absent from commonly used culture media. Mimicking physiologic nutrient conditions has profound effects on cellular metabolism, and analysis of tumor metabolism with stable isotope-labeled metabolites in HPLM can reveal metabolic activities that may be obscured by standard cell culture media.^3^^,^^27^ However, efforts to use in vitro glioma models with physiologic media remain limited, potentially due to concerns that altered nutrient conditions may induce GSC differentiation or death. Therefore, it is not clear whether HPLM culture can be exploited to unmask interactions between metabolism and the immune microenvironment in glioma, as has been demonstrated in other contexts.^28^

One promising system for modeling glioma metabolism in vitro is the use of surgically explanted glioma tissue, particularly Surgically eXplanted Organoids (SXOs). Surgically explanted organoids are efficiently created without single-cell dissociation of the resected tumor tissue, allowing maintenance of local cytoarchitecture.^30^ These models recapitulate parental tumor features such as cellular heterogeneity, cell-cell and cell-stroma interactions, gene expression and mutational profiles, and treatment response.^30–32^ While SXOs offer advantages over conventional models, they are typically cultured in non-physiologic media such as Glioma Organoid Complete (GOC) medium, likely altering nutrient-sensitive metabolic programs. Integrating HPLM culture with glioma organoid modeling efforts may improve fidelity of metabolism studies while retaining influences of the native tumor microenvironment.

Here, we integrate adapted formulations of HPLM with stable isotope tracing in both GSC monocultures and IDH-wildtype GBM tissue explants. We develop a quantitative labeling score to compare metabolic pathway activity between models and pair these data with spatial transcriptomics to identify nutrient-sensitive transcriptional programs. This approach establishes a physiologic in vitro model of glioma metabolism that complements existing strategies to study tumor cell-intrinsic and microenvironmental metabolic activities.

## Methods

### Human Subjects

This study was conducted according to the principles of the Declaration of Helsinki. Patient tissue and blood were collected following ethical and technical guidelines on the use of human samples for biomedical research at UT Southwestern Medical Center or the University of Pittsburgh after informed patient consent under protocols approved by an IRB (STU 022011-070, STU 092014-026, or STUDY19080321).

### Cell Culture

Detailed information on cell lines and culture conditions is included in Supplementary Methods. All lines were cultured at 5% CO2, ambient oxygen, and 37 °C. All lines were routinely evaluated for mycoplasma contamination and confirmed to be negative.

### Cloning, Transfection, and Viral Transduction

Dihydropyrimidine dehydrogenase (DPYD) and empty vector expression constructs were generated via Gateway cloning (Thermo Fisher). Lentivirus was produced in HEK293T cells and used to transduce HOG cells. Stable cell lines were selected in 2 µg/mL puromycin (GoldBio P-600-100).

### Explant SXO Creation and Culture in GOC

Explant SXO cultures were generated as described previously.^32^ Stocks of GOC were used within a maximum of 1 week after preparation. Plates were rotated at 120 rpm in a humidified incubator at 37 °C, 5% CO_2_, and 21% oxygen. Glioma organoid complete was replaced in SXO cultures every 48 hours.

### Derivation of GSCs from SXOs

Surgically explanted organoids were derived as described above. Surgically explanted organoids tissue was dissociated to form GSCs using the Brain Tumor Dissociation Kit (Miltenyi Biotech 130-095-942) according to the manufacturer’s instructions. Derived GSCs were cultured in GOC for 24 hours, then for 24 hours in a mixture of 50% GOC and 50% SXO HPLM, then for 120 hours in 100% SXO HPLM (changing media every 24 hours).

### Culture in GSC HPLM

Human plasma-like medium was prepared as previously described^27^^,^^28^^,^^33^ but without the addition of dialyzed fetal bovine serum and glutamate. Supplementation and stable isotope tracer incorporation into GSC HPLM are described in Supplementary Methods.

### Explant Culture in SXO HPLM

Human plasma-like medium was prepared as previously described^27^^,^^28^^,^^33^ but without addition of dialyzed fetal bovine serum. Supplementation and stable isotope tracer incorporation into SXO HPLM are described in Supplementary Methods.

### 5-Fluorouracil Treatment and Flow Cytometry

HOG cells treated with 5-FU or DMSO were stained with AnnexinV-FITC and DAPI and analyzed on an LSR Fortessa flow cytometer (BD Biosciences).

### Histology and Immunohistochemistry

Embedding, sectioning, histology, immunohistochemistry, and image acquisition were performed by HistoWiz, Inc. (histowiz.com).

### Immunoblotting

Protein lysates were resolved by SDS-PAGE and transferred to nitrocellulose membranes (Bio-Rad 1620112) prior to incubation with primary antibodies against DPYD, FLAG, or glyceraldehyde 3-phosphate dehydrogenase (GAPDH).

### Liquid Chromatography-Mass Spectrometry

Metabolite extraction is described in Supplementary Methods. Liquid Chromatography-Mass Spectrometry was performed as described previously.^34^^,^^35^ Briefly, 10 µL of SXO metabolite extract or 20 µL of GSC metabolite extract was injected and analyzed with a Q Exactive HF-X or Exploris orbitrap mass spectrometer (Thermo Fisher) coupled to a Vanquish ultra-high performance liquid chromatography system (Thermo Fisher).

### Derivation of Metabolite Labeling Score

Total fractional enrichment of all quantified metabolites was calculated. Metabolites were filtered for total labeling > 0 and adequate total pool size. Each metabolite’s total labeling was divided by label accumulation in glutamate to normalize and derive the Metabolite Labeling Score.

### Metabolite Set Enrichment Analysis

Quantitative enrichment analysis was performed using MetaboAnalyst (6.0).^36^^,^^37^ Metabolite Labeling Scores were compared between models without normalization, filtering, or scaling.

### DSP Assay

Spatial expression profiles were analyzed using GeoMx DSP (NanoString Technologies, RRID: SCR_021660). SYTO-13, GFAP, and CD45 were used as visualization markers. Gene expression analysis was performed using GeoMx Analysis suite (2.4.2.2, RRID: SCR_023424).

### Gimeracil and TNFα Treatment

Gimeracil (Cayman Chemical 16525) was used at 30 µM and TNFα (Millipore Sigma H8916) was used at 10 ng/mL where indicated.

### CD44 Quantification

Cells were stained with CD44-FITC (Miltenyi 130-113-341) and CD24-APC (Miltenyi 130-095-954) or the isotype controls IgG1-FITC (Miltenyi 130-113-437) and IgG1-APC (Miltenyi 130-113-196) according to manufacturer’s instructions. Cells were analyzed using an LSR Fortessa (BD Biosciences) flow cytometer.

### TCGA Expression Analysis

The TCGA GBM^38^^,^^39^ dataset was used to assess *CD44* and *DPYD* expression in human gliomas (https://www.cbioportal.org).

### Quantification and Statistical Analysis

Statistical analyses were performed with GraphPad Prism (10.6.1, RRID: SCR_002798) and included both descriptive statistics as well as tests of statistical significance. All data are plotted as mean ± SEM. For all tests, *P-*values less than 0.05 were considered statistically significant.

## Results

### HPLM Preserves Glioma Cytoarchitecture and Reveals Nutrient-Responsive Transcriptional Programs

**Figure 1.**
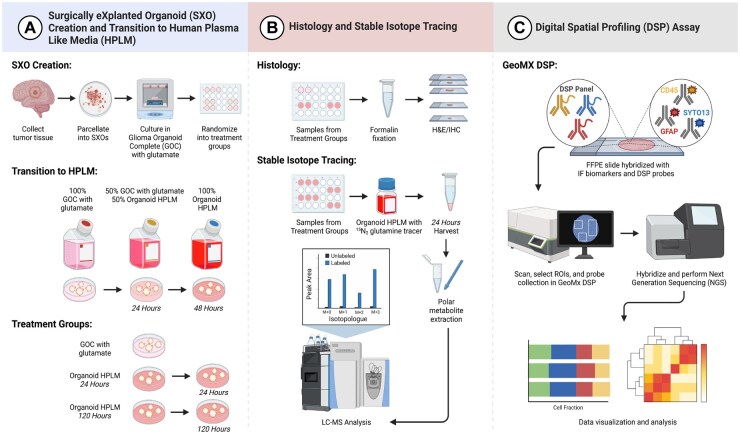
Experimental overview. (A) Glioblastoma Surgically eXplanted Organoid (SXO) creation and transition to Human Plasma-Like Medium (HPLM). Human brain tumor tissue was collected directly from the operating room, manually parcellated, and cultured as explants. Surgically explanted organoids were cultured in Glioma Organoid Complete Medium with glutamate (GOC), then randomized to culture in GOC or SXO HPLM. For HPLM culture, SXOs were first transferred into media containing 50% GOC and 50% SXO HPLM for 24 hours, then preconditioned in 100% SXO HPLM for either an additional 24 or 120 hours. (B) Histology and stable isotope tracing. *Histology:* SXOs were fixed in neutral buffered formalin, then were stained for hematoxylin and eosin (H&E), Ki67, and SOX2. *Stable Isotope Tracing*: SXOs cultured in SXO HPLM were transferred into SXO HPLM containing ^15^N_2_-glutamine. After 24 hours, polar metabolites were harvested and subjected to liquid chromatography-mass spectrometry (LC-MS). (C) NanoString Digital Spatial Profiling (DSP) Assay. Formalin-fixed paraffin-embedded tissue slides were deparaffinized and stained for relevant markers (SYTO-13 to identify DNA, GFAP to identify tumor cells, and CD45 to identify immune cells). Spatially resolved regions of interest (ROIs) were selected for analysis based on fluorescent images. Oligo-tagged probes for each ROI were used for spatial transcriptomics analysis. Figure created with BioRender (www.biorender.com).

We developed an approach to leverage HPLM culture and SXO glioma models to study glioma metabolism (**Figure 1**). Our approach included comparisons of explants cultured in conventional GOC medium with those grown in HPLM. We conducted histological, metabolic, and spatial transcriptomic analyses of glioma SXOs and benchmarked our findings against GSC monocultures to discriminate tumor cell-intrinsic and -extrinsic properties reflected in explant models. To do so, we established SXOs from surgically resected IDH-wildtype glioblastoma (GBM) tissues (**Table S1**).

We first compared the viability and cytoarchitecture of glioma explants cultured in an SXO-adapted formulation of HPLM to those cultured in conventional GOC medium (**Figure 2A-F**). Histological evaluation by a board-certified neuropathologist (TER) revealed that explants maintained hallmark features of GBM—including necrosis, microvascular proliferation, high mitotic index, and cellular atypia—regardless of culture condition. Quantitative analysis of nuclei demonstrated that SXOs cultured in SXO HPLM for 24 or 120 hours maintained similar cell density to those cultured in GOC (**Figure 2G**). These results suggest that explants do not acutely lose integrity or cellularity upon transition to HPLM culture. We assessed Ki67 expression (**Figure 2H-M**) and found that SXOs cultured in GOC or SXO HPLM for 24 hours exhibited similar proliferation, while the SXOs cultured in SXO HPLM for 120 hours exhibited a slight proliferation decrease (**Figure 2N**). We also evaluated explant tissues for expression of the stemness marker SOX2 (**Figure 2O-T**). SOX2-positive cell frequencies were similar between all groups (**Figure 2U**), suggesting that HPLM culture does not cause GSC differentiation over five days.

**Figure 2.** Surgically explanted organoids (SXO) histology and transcriptional response after culture in SXO HPLM. (A-F) H&E of representative SXOs cultured in (A) Glioma organoid complete (GOC), (B) SXO HPLM for 24 hours, and (C) SXO HPLM for 120 hours, which are shown at high magnification in (D-F). Scale bars = 100 μm. (G) Imaging-based quantification of cell density by evaluation of nuclei/µm^2^. ns = not significant (Tukey’s multiple comparisons test). (H-M) Ki67 immunohistochemistry (IHC) of representative SXOs cultured in (H) GOC, (I) SXO HPLM for 24 hours, and (J) SXO HPLM for 120 hours, which are shown at high magnification in (K-M). Scale bars = 100 μm. (N) Quantification of Ki67-positive cells. ns = not significant, *P < 0.05 (Dunnett’s T3 multiple comparisons test). (O-T) SOX2 IHC of representative SXOs cultured in (O) GOC, (P) SXO HPLM for 24 hours, and (Q) SXO HPLM for 120 hours, which are shown at high magnification in (R-T). Scale bars = 100 μm. (U) Quantification of SOX2-positive cells. ns = not significant (Dunnett’s T3 multiple comparisons test). (V-X) NanoString morphology scans demonstrating immunofluorescent staining for SYTO-13 (nuclei, blue), GFAP (tumor and glial cells, red), and CD45 (immune cells, yellow) in representative SXOs cultured in (V) GOC, (W) SXO HPLM for 24 hours, and (X) SXO HPLM for 120 hours. Scale bars = 100 μm. (Y) CIBERSORT imputed cell fractions from NanoString gene expression data. (Z) Variant allele frequencies for identified variants of strong clinical significance and variants of possible clinical significance by a clinical next-generation sequencing assay using DNA and RNA sequencing of FFPE of parental tumor for SXO210 compared to patient germline DNA from saliva. (AA) Gene set enrichment analysis (GSEA) of geometric mean normalized NanoString sequencing data from SXO210 cultured in SXO HPLM for 120 hours versus SXO210 cultured in GOC, grouped by pathway category. *P* values are determined by GSEA. (BB) NanoString morphology scan in a representative immune-segmented SXO210 ROI, with highlights demonstrating segmentation (CD45^low^ red, CD45^high^ green). Scale bar = 100 μm. (CC) Quantification of relative CD69 expression in CD45^high^ segments of SXO210 ROIs. Expression data are geometric mean normalized and *z-*scored relative to other genes. ns = not significant, *P < 0.05 (unpaired *t*-test). Data are presented as means ± SEM.

To evaluate cellular heterogeneity and gene expression programs, we performed NanoString-based spatial transcriptomics of SXOs cultured in GOC or SXO HPLM for 24 or 120 hours using immunofluorescent markers SYTO-13 (to mark DNA), GFAP (to mark astrocytes and malignant cells), and CD45 (to mark immune populations) (**Figure 2V-X**). Morphology scans for NanoString analysis were collected and used to select regions of interest (ROIs) containing either diverse heterocellular populations or predominantly immune cells for spatially resolved sequencing. Using sequencing results from heterocellular SXO ROIs, we employed the CIBERSORT algorithm^40^ to impute cell identities and estimate fractions of cell types present in each sample (**Figure 2Y**). Surgically explanted organoids contained diverse, spatially variable cell populations, consistent with prior work.^30^^,^^32^ Cellular composition of SXOs did not vary between culture conditions. Sequencing of the parental tumor used to generate SXOs revealed mutations of likely clinical significance affecting *TP53*, *RB1*, *PTEN*, *NF1*, and *TERT* genes (**Figure 2Z**). Using NanoString sequencing results, we performed gene set enrichment analysis. Surgically explanted organoids cultured in HPLM versus GOC medium demonstrated differential expression of genes related to translation, gene transcription, cellular metabolism, the cell cycle, and immunologic pathways (**Figure 2AA** and **Table S2**).

Prior work indicates that HPLM culture promotes T cell activation compared to conventional medium.^28^ Therefore, we sought to understand how HPLM culture affects gene expression programs in immune cells. CD45 is expressed on microglia and nucleated hematopoietic cells, including macrophages and lymphocytes.^41^ We classified SXO regions as CD45^high^ or CD45^low^ (**Figure 2BB**) to evaluate how physiologic nutrient conditions affect gene expression programs in immune cell-enriched and immune cell-depleted tumor environments. Gene sets related to interleukin and interferon signaling and immune cell activation were enriched in CD45^high^ compartments of SXOs grown in HPLM versus GOC (**Table S3**). We also used sequencing of CD45^high^ regions to investigate expression of CD69, a marker of T-cell activation.^42^ CD69 expression increased in a time-dependent manner following transition to HPLM culture (**Figure 2CC**). We systematically evaluated differences in nutrient composition between HPLM and GOC that may contribute to these effects on immune cells (**Table S4**). Levels of formate, taurine, and lactate differed significantly between these medium types and have been linked to regulation of immune cell priming, activation, and behavior.^43–45^ Together, these data indicate that SXOs cultured in HPLM maintain viability, recapitulate the cellular heterogeneity of parental tumors, engage transcriptional programs that are dampened in conventional tissue culture media, and display heightened immune cell activity.

### Stable Isotope Tracing in HPLM to Compare Metabolic Patterns in GSC Monocultures and Glioma Explants

To assess the effects of physiologic nutrient conditions on metabolic states of explant cultures, we performed metabolomics analysis of GBM tissue snap-frozen upon resection and of SXOs (model SXO382, **Table S1**) generated from this tissue and subsequently cultured in HPLM or GOC medium. Human plasma-like medium-cultured explants maintained metabolite levels more similar to parental tumor tissue relative to SXOs grown in GOC (**Figure S1A**). We also observed that HPLM culture of another GBM explant (model SXO512, **Table S1**), prevented reverse urea cycle flux that has been shown to occur in medium preparations with supraphysiologic arginine content,^29^ including GOC (**Figure S1B** and **Table S4**). Importantly, HPLM culture normalized levels of urea cycle intermediates arginine and citrulline in SXOs (**Figure S1C and D**), as well as the ratio of argininosuccinate to citrulline (**Figure S1E**). Moreover, ^15^N_2_-glutamine stable isotope tracing showed increased labeling of argininosuccinate and arginine in HPLM-grown SXOs versus GOC-grown SXOs (**Figures S1F and G**), indicative of appropriate forward flux through the urea cycle in the former.

After establishing superior preservation of metabolic activities in SXOs grown in HPLM versus GOC medium, we next analyzed global ^15^ N_2_-glutamine stable isotope tracing patterns in explants cultured in SXO HPLM. SXO210 organoids were preconditioned in tracer-free HPLM for either 24 or 120 hours and then transferred to tracer-free or ^15^ N_2_-glutamine-containing HPLM for 24 hours (**Figure 3A**). Regardless of the length of preconditioning employed, stable isotope tracing experiments in this study were performed over 24 hours. We employed LC-MS to quantify ^15^N-labeled and unlabeled metabolites in extracts from these organoids. First, we confirmed intra-organoid accumulation of isotopic label in glutamine (**Figure 3B**). We compared label accumulation from ^15^ N_2_ glutamine in all metabolites with detectable labeling between SXOs preconditioned for 24 and 120 hours in HPLM. Importantly, we observed minimal metabolite labeling in explants cultured in tracer-free HPLM (**Figure 3B**). Linear regression analysis revealed minimal differences in metabolite labeling between SXOs preconditioned for 24 or 120 hours in HPLM prior to tracing, with an *r*^2^ of 0.9427 indicating strong concordance between conditions (**Figure 3C**). Among select metabolites representing a diverse array of biosynthetic pathways (including nonessential amino acid biosynthesis, branched-chain amino acid transamination, redox homeostasis, nucleotide synthesis, and the urea cycle), there were no significant differences in labeling between 24- and 120-hour preconditioned SXOs (**Figure 3D**). Therefore, we preconditioned all SXOs for 120 hours in HPLM prior to tracing in all subsequent studies.

**Figure 3.** Stable isotope tracing in Surgically explanted organoids (SXOs) and GSCs reveals tumor cell-intrinsic and -extrinsic features of glioma metabolism. (A) Schema depicting timeline of SXO tracing experiments in HPLM, with both conditions exposed to tracer for 24 hours. (B-D) ^15^N_2_-glutamine (Gln) stable isotope tracing assay in SXO210. *n *= 2 for unlabeled and 24-hour HPLM preconditioning groups, *n *= 3 for 120-hour HPLM preconditioning group. (B) Fractional enrichment (FE) of the M + 2 isotopologue of glutamine in glutamine pool. *P < 0.05 (one-way ANOVA). (C) Correlation between label accumulation in all detected and labeled metabolites between 24-hour and 120-hour HPLM preconditioning. *r* = Pearson’s correlation coefficient. *P* value was determined by simple linear regression analysis. (D) Total FE (1—M + 0 isotopologue FE) of label from ^15^N_2_ glutamine in intracellular metabolite (y-axis) pools in each culture condition (x-axis). ns = not significant (unpaired *t*-test for each metabolite). (E) Schema depicting derivation of Metabolite Labeling Score. (F-H) Volcano plots of Metabolite Labeling Scores in SXO210 compared with Metabolite Labeling Scores in GSC lines (F) UTSW63, (G) TS516, and (H) HK157. In all SXOs and GSCs, ^15^N_2_-glutamine tracing was conducted for 24 hours. Horizontal lines represent *P* value of 0.05, vertical lines represent fold-changes of 1.25. Two-tailed *P* values were determined by unpaired *t*-test. (I-K) Metabolite Set Enrichment Analysis of differentially labeled metabolites in SXO210 versus (I) UTSW63, (J) TS516, and (K) HK157 GSC lines. *P* values were determined by the quantitative enrichment analysis algorithm. Data are presented as mean ± SEM. Figure created with BioRender (www.biorender.com).

To compare metabolic activities of heterocellular glioma explants with GSC monocultures, we performed ^15^ N_2_-glutamine stable isotope tracing in HPLM in one GBM explant model (model SXO210, **Table S1**) and three GBM GSC models: UTSW63, TS516, and HK157. As comparison of label enrichment between SXOs and GSCs may be compromised by differences in proliferation, de novo glutamine synthesis rates, tissue-specific intercellular metabolic cycles, and other factors, we developed a Metabolite Labeling Score to facilitate comparisons across model systems (**Figure 3E**). The Metabolite Labeling Score uses natural isotope abundance-corrected fractional labeling information, applies quality filters, and then normalizes to the level of label accumulation from glutamine in glutamate. Comparison of tracing patterns between SXO210 and each of the three GSC lines revealed differential labeling of multiple classes of metabolites, including purine nucleobases, guanosine diphosphate mannose (GDP-mannose), and pyrimidine degradation products (**Figure 3F-H**). Applying Metabolite Set Enrichment Analysis (MSEA) to differentially labeled metabolites between SXO210 and each GSC line, we identified several pathways commonly altered in explants, including purine metabolism and pyrimidine metabolism (**Figure 3I-K**). Together, these data indicate that stable isotope tracing in SXOs may enable identification of TME-dependent metabolic phenotypes not readily identified in GSC monocultures.

### Immune-Modulatory Purine Synthesis is a Tumor Cell-Intrinsic Feature of Glioma Metabolism

We observed that glutamine-dependent labeling of various purine metabolites (**Figure 4A**) differed between explants and GSCs. Compared to SXO210, the GSCs UTSW63, TS516, and HK157 demonstrated high labeling in adenosine and hypoxanthine (**Figure 4B and C**), indicating that degradation of purine nucleotides generated via the de novo purine synthesis pathway is enhanced in tumor cells. Purine nucleotides, such as adenosine monophosphate (AMP) and guanosine monophosphate (GMP), can be synthesized de novo through a multi-enzyme process reliant on glutamine as a nitrogen donor.^46^ Purine nucleotides can also be degraded to form nucleosides and nucleobases, including adenosine and hypoxanthine.^46^ Extracellular adenosine acts as an important immunosuppressive metabolite through its binding to adenosine receptors on multiple cell types.^46–48^ In glioma, accumulation of adenosine through secretion or extracellular degradation of adenosine triphosphate (ATP) has been implicated in immune suppression and tumor progression.^46^ Elevated glutamine-dependent synthesis of adenosine in GSCs could not be explained by higher rates of de novo purine synthesis in these cells, as glutamine labeling of the nucleotide AMP was similar (**Figure 4D**) and GMP was higher (**Figure 4E**) in SXOs relative to GSCs. These data indicate that tumor cells make substantial contributions to immunosuppressive adenosine synthesis in the glioma TME. Further, our findings show that comparing metabolic patterns in glioma explants and GSCs can be used to determine glioma cell-intrinsic contributions to brain tumor metabolism.

**Figure 4.** Stable isotope tracing in surgically explanted organoids (SXOs) captures tumor cell-intrinsic immune-modulatory purinergic metabolism. (A) Schema of glutamine-dependent synthesis of purine nucleotides and their degradation products. IMP = inosine monophosphate. AMP = adenosine monophosphate. GMP = guanosine monophosphate. (B-E) ^15^N_2_ glutamine stable isotope tracing assays in SXO210 explants and UTSW63, TS516, and HK157 GSC lines. Tracing duration was 24 hours. Metabolite Labeling Scores for purine degradation products (B) adenosine and (C) hypoxanthine and for purine nucleotides (D) adenosine monophosphate (AMP) and (E) GMP are shown. *n *= 3 for all groups. *P < 0.05, ***P < 0.001 (one-way ANOVA). Data are presented as mean ± SEM.

### Tracing Studies in Glioma Explants Reveal Stromal Production of Glycosylation Substrates

**Figure 5.**
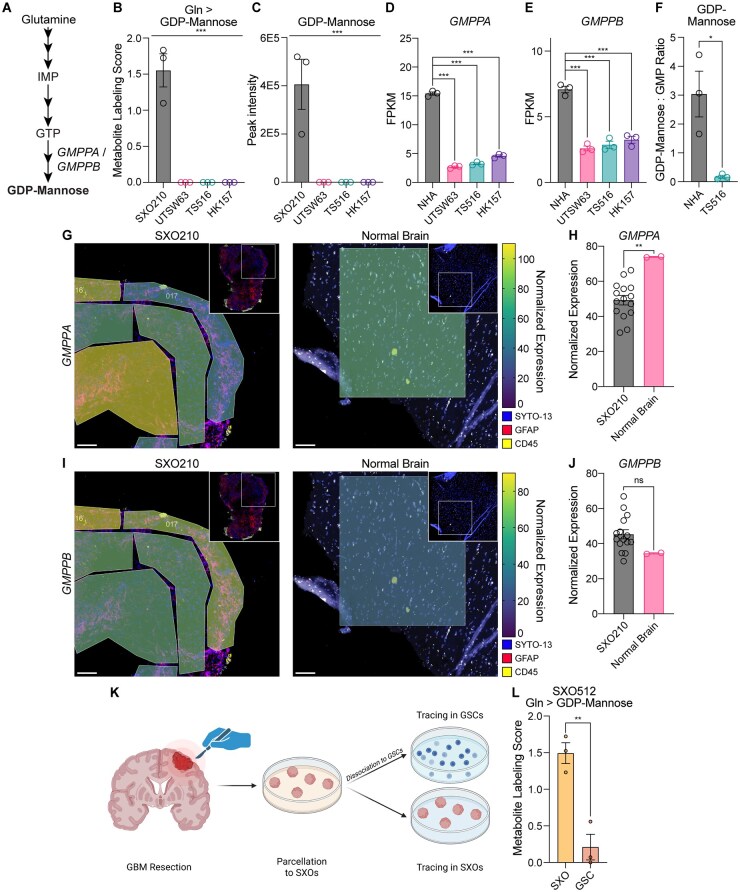
Surgically explanted organoids (SXO) tracing reveals GDP-mannose synthesis by stromal astrocytes in the glioma TME. (A) Schema of de novo purine and guanosine diphosphate-mannose (GDP-mannose) synthesis pathways. IMP = inosine monophosphate. GTP = guanosine triphosphate. (B) ^15^N_2_ glutamine stable isotope tracing assays in SXO210 explants and UTSW63, TS516, and HK157 GSCs. Tracing duration was 24 hours. Metabolite Labeling Scores for GDP-mannose are shown. *n *= 3 for all groups. ***P < 0.001 (one-way ANOVA). n.d. = not detected. (C) Steady-state quantification of GDP-mannose. *n *= 3 for all groups. **P < 0.01 (one-way ANOVA). n.d. = not detected. (D-E) RNA sequencing of NHA immortalized astrocytes and GSC lines, representing fragments per kilobase of transcript per million mapped reads (FPKM) for (D) *GMPPA* and (E) *GMPPB* genes. *n *= 3 for all groups. ∗∗∗*P *< 0.001 (unpaired *t*-test). (F) Abundance of GDP-Mannose normalized to GMP abundance in NHA and TS516 cells. *n *= 3. *P < 0.05 (unpaired *t*-test). (G-J) Heatmap of (G) *GMPPA* or (I) *GMPPB* expression by geometric mean normalized NanoString sequencing data in representative ROIs of SXO210 explants and normal human brain tissue. Scale bar = 100 μm. Quantification of geometric mean normalized expression in all ROIs of (H) *GMPPA* and (J) *GMPPB*. Data are presented as mean ± SEM. (K) Schema depicting paired SXO and GSC tracing experiment. (L) ^15^N_2_ glutamine stable isotope tracing assays in SXO512 explants or SXO512-derived GSCs. Tracing duration was 24 hours. Metabolite labeling scores for GDP-mannose are shown. **P < 0.01 (unpaired *t*-test). Figure created with BioRender (www.biorender.com).

We also found that glutamine-dependent GDP-mannose synthesis was consistently downregulated in GSCs compared to SXOs (**Figures 3F-H and 5A and B**). Guanosine diphosphate-mannose (GDP-mannose) is the major mannosyl donor for glycosylphosphatidylinositol anchor synthesis, O-mannosylation, and N-linked glycosylation. Therefore, our data implied that stromal cells in the glioma TME may play an important role in generating metabolic substrates for protein glycosylation. *GMPPA* and *GMPPB* encode GDP-mannose pyrophosphorylase enzymes, which catalyze the conversion of the purine nucleotide guanosine triphosphate and mannose-1-phosphate to GDP-mannose (**Figure 5A**). GDP-mannose was not detectable in GSCs, while a peak was detectable and quantified in SXO210 (**Figure 5C**). We analyzed our previously published dataset of RNA sequencing in the three GSC lines^49^ and added analysis of NHA immortalized, nonmalignant astrocytes. *GMPPA* and *GMPPB* were more highly expressed in NHAs relative to GSCs (**Figure 5D and E**), suggesting that stromal astrocytes drive GDP-mannose synthesis in glioma. Indeed, steady-state levels of GDP-mannose were much higher in NHA astrocytes versus TS516 GSCs (**Figure 5F**). Both *GMPPA* (**Figure 5G and H**) and *GMPPB* (**Figure 5I and J**) were robustly expressed in SXO210 and normal human brain tissues. To directly assess the contribution of stromal GDP-mannose synthesis in explant cultures, we collected a GBM specimen and parcellated it to SXOs (model SXO512, **Table S1**), maintaining half as explants and dissociating the other half to GSC monocultures (**Figure 5K**). ^15^ N_2_-glutamine stable isotope tracing in these paired cultures retaining and lacking stromal cells, respectively, demonstrated robust GDP-mannose labeling in explants but not purified tumor cell cultures (**Figure 5L**). Taken together, these data establish that comparative stable isotope tracing in glioma explant and GSC models can be used to identify tumor cell-extrinsic metabolic activities in the glioma TME.

### Pyrimidine Degradation Reflects the Mesenchymal Cell State in Glioma

**Figure 6.**
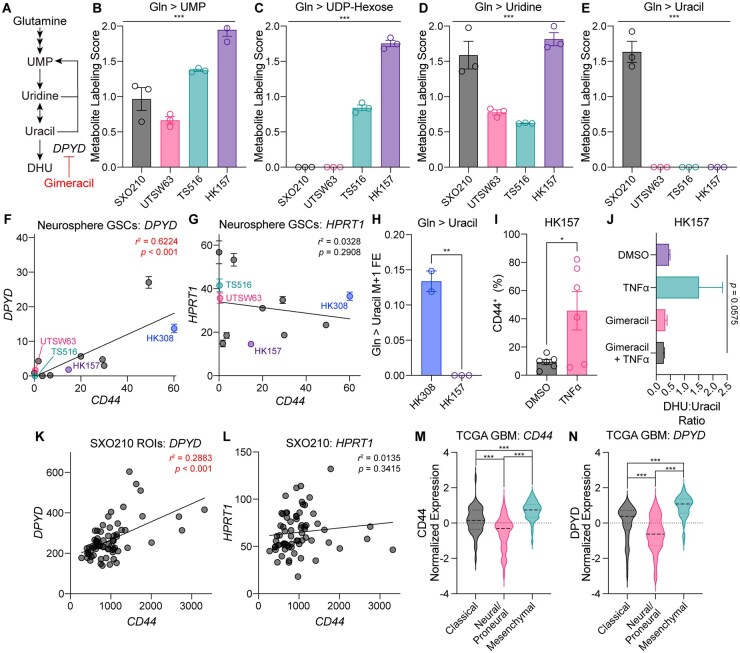
Metabolic features of mesenchymal GBMs. (A) Schema of pyrimidine synthesis and degradation pathways. UMP = uridine monophosphate. DHU = dihydrouracil. (B-E) ^15^N_2_ glutamine stable isotope tracing assays in SXO210 explants and UTSW63, TS516, and HK157 GSC lines. Tracing duration was 24 hours. Metabolite Labeling Scores for (B) UMP, (C) UDP-hexose, (D) uridine, (E) and uracil are shown. *n *= 3 for all groups. ***P < 0.001 (one-way ANOVA). (F-G) Correlation between expression of *CD44* and (F) *DPYD* or (G) *HPRT1* in the GSC lines BT054, HK157, HK211, HK252, HK308, MGG152, TS516, TS603, UTSW5, UTSW63, and UTSW71 by RNA sequencing. Expression values are shown in Fragments Per Kilobase of transcript per Million mapped reads (FPKM). *r* = Pearson’s correlation coefficient. *P* values were determined by simple linear regression. *n *= 3 per line. (H) Amide-^15^N glutamine stable isotope tracing for 18 hours in HK308 and HK157 GSC lines. *n *= 2 in HK308, *n *= 3 in HK157. **P < 0.01 (unpaired *t*-test). FE = fractional enrichment. (I) Flow cytometry quantification of CD44 in HK157 cells treated for 7 days with DMSO or TNFα. *P < 0.05 (unpaired *t*-test). (J) Ratio of DHU to uracil in HK157 cells treated for 7 days with DMSO, 10 ng/mL TNFα, 30 µM gimeracil, or 10 ng/mL TNFα and 30µM gimeracil. *P* value was determined by one-way ANOVA. (K-L) Correlation between geometric mean normalized expression of *CD44* and (K) *DPYD* or (L) *HPRT1* in SXO210 ROIs by NanoString sequencing. *P* values were determined by simple linear regression. (M-N) Expression of (M) *CD44* or (N) *DPYD* in The Cancer Genome Atlas (TCGA) GBM dataset by transcriptional subtype. ***P < 0.001 (unpaired *t*-test). Results shown here are based on data generated by the TCGA Research Network from Verhaak et al.^39^ Expression values are log_2_ transformed, median centered, and scaled by median absolute deviation. In (B-E) and (H-J), data are presented as mean ± SEM.

Pyrimidine nucleotides represent another class of metabolites that we observed to be differentially labeled by glutamine in SXO and GSC models (**Figures 3F-K and 6A**). Several lines of evidence led us to hypothesize that this difference may be related to specific glioma cell transcriptional states. GBMs contain cells arising from distinct genetic subclones that display several recurrent transcriptional phenotypes, including a mesenchymal type associated with loss-of-function mutations in the *NF1* gene.^50^ Previous work has linked pyrimidine degradation pathway activity with mesenchymal cell identity in other cancer contexts.^51^ Given that the SXO210 model was derived from a GBM with an *NF1* loss-of-function mutation^52^ (**Figure 2Z**), we hypothesized that SXO210 organoids may be enriched for mesenchymal-type glioma cells that actively degrade pyrimidine nucleotides produced by the glutamine-dependent de novo synthesis pathway. The pyrimidine nucleotide uridine monophosphate (UMP) can be synthesized de novo from glutamine, aspartate, and bicarbonate or salvaged from uridine.^46^ Pyrimidine degradation occurs through multiple steps, the first of which involves conversion of uridine to uracil. Uracil can then be converted to dihydrouracil (DHU) by dihydropyrimidine dehydrogenase, an enzyme encoded by *DPYD.*^46^ To test our hypothesis, we first evaluated glutamine-dependent UMP synthesis. SXO210 and GSC cultures exhibited similar glutamine label accumulation in UMP, indicating comparable rates of de novo pyrimidine synthesis (**Figure 6B**). In contrast, glutamine labeling of pyrimidines downstream of UMP displayed substantial heterogeneity across models. Label from glutamine variably accumulated in UDP-hexose (**Figure 6C**), while all models exhibited label accumulation in the pyrimidine salvage substrate uridine (**Figure 6D**). While label from glutamine was present in uridine across all lines, only SXO210 exhibited label accumulation in uracil (**Figure 6E**). This result indicated that only the SXO210 model displayed robust pyrimidine degradation. Consistent with the idea that this metabolic activity may be driven by mesenchymal glioma cells enriched in SXO210 explants, the UTSW63, TS516, and HK157 GSC lines expressed low or undetectable levels of the mesenchymal marker CD44 (**Figure 6F**).

To investigate whether pyrimidine degradation is associated with the mesenchymal subtype of GBM, we leveraged an RNA sequencing dataset of GSC lines.^49^ We found a strong correlation between expression of the mesenchymal marker *CD44* and *DPYD* but not between *CD44* and *HPRT1*, a gene encoding an enzyme involved in purine metabolism (**Figure 6F and G**). Using GSC lines with high (HK308) or low (HK157) *CD44* expression, we performed amide-^15^ N glutamine stable isotope tracing and confirmed that glutamine label accumulated in uracil in mesenchymal HK308 cells but not non-mesenchymal HK157 cells (**Figure 6H**). Tumor necrosis factor-alpha (TNFα) is an inflammatory cytokine that can induce epithelial-to-mesenchymal transition.^53^ Treating HK157 cells with TNFα induced mesenchymal identity, as marked by CD44 upregulation (**Figure 6I**). Treating HK157 cells with TNFα also increased the ratio of DHU to uracil, a metabolic marker of pyrimidine degradation,^51^ which was reversed by the DPYD inhibitor gimeracil (**Figure 6J**). These findings indicate that mesenchymal differentiation of GSCs is sufficient to activate pyrimidine degradation.

Deficiencies in DPYD activity cause sometimes-fatal hypersensitivity to the chemotherapeutic agent 5-fluorouracil (5-FU).^54^ To test whether an inverse relationship may exist that renders glioma cells with higher levels of DPYD less sensitive to 5-FU treatment, we generated isogenic glioma stable lines expressing FLAG-tagged DPYD or an empty vector (EV) control (**Figure S2A**) and treated them with 5-FU (**Figure S2B**). Consistent with this hypothesis, cells expressing DPYD exhibited decreased cell death upon treatment with 5-FU. These data suggest that studying glioma explant metabolism under physiologic nutrient conditions may be useful in identifying relationships between metabolic programs and treatment responses.

Intratumoral heterogeneity of transcriptional phenotypes is a common feature of GBMs,^50^ so we examined the association of the mesenchymal phenotype with *DPYD* expression in individual ROIs of SXO210 explants via spatial transcriptomics. Regions with higher *CD44* expression were associated with higher expression of *DPYD* (**Figure 6K**), but not *HPRT1* (**Figure 6L**). To test whether this association was broadly relevant in glioma, we turned to The Cancer Genome Atlas studies of GBM.^38^^,^^39^ Consistent with our cell culture and SXO studies, expression of both *CD44* (**Figure 6M**) and *DPYD* (**Figure 6N**) were elevated in GBMs determined to display the mesenchymal transcriptional subtype by gene signature analysis. Taken together, our data establish that metabolic hallmarks of GBM transcriptional subtypes are reflected in stable isotope tracing assays of glioma explants.

## Discussion

Recent advances in mass spectrometry technology and metabolite probe development have produced deeper understanding of tumor metabolism. In glioma, the discovery of recurrent hotspot mutations in *IDH1* and *IDH2* genes^55^^,^^56^ has provided added rationale to investigate the association between altered metabolism and oncogenesis. Investigation of tumor-intrinsic metabolism in glioma has yielded insights into therapeutic vulnerabilities, including de novo pyrimidine synthesis,^3^^,^^4^^,^^7^^,^^17^ purine synthesis,^8^^,^^57^ glutathione metabolism,^9^^,^^10^ dopaminergic signaling,^11^ malate dehydrogenase activity,^12^ and threonine metabolism.^13^ Glioma cells further have been shown to alter cell-intrinsic nucleotide metabolism to mediate chemo- and radio-resistance.^14–16^^,^^57^^,^^58^ Additional research has revealed critical contributions of intercellular crosstalk in the TME to glioma biology and metabolism. For example, altered metabolism of glioma cells has been linked to epigenetic rewiring that suppresses antitumor immunity^59^^,^^60^ and TME lymphatic endothelial-like cells have been shown to regulate glioma cell cholesterol metabolism.^61^

Steady-state metabolomics analyses of primary tissue samples have provided key insights into TME-dependent contributions to glioma metabolism.^62–65^ These studies measure total metabolite levels but are limited in their ability to assess metabolic pathway activity. In comparison, stable isotope tracing techniques offer the ability to monitor dynamic metabolic processes. Intraoperative stable isotope tracer infusions in patients with gliomas have been reported using ^13^C_6_-glucose or hyperpolarized ^13^ C-labeled metabolite probes.^20–23^ These techniques, however, are expensive, may require perioperative intervention, involve intricate coordination between clinicians and researchers, and typically investigate a single metabolite tracer per patient.

Studies of metabolism involving banked tissue specimens or tracer infusions in patients are complemented by cell culture and murine disease models. Employing such models obviates the need for clinical coordination and creates opportunities to apply multiplexed or high-throughput approaches to assess tumor metabolism. However, conventional cell culture models often lack the metabolic and cellular complexity of the tumor microenvironment (TME), and common incorporation of non-physiologic nutrient conditions limits translational relevance. While orthotopic murine models address some of these limitations, they, too, are technically challenging and expensive—particularly for isotope tracing—and are constrained by the use of immunocompromised hosts, precluding study of immune-metabolic crosstalk. These challenges underscore the need for tractable, physiologically faithful ex vivo models that recapitulate the metabolic and immunologic context of human gliomas.^31^

We present a method that integrates defined, physiologic culture conditions with stable isotope tracing to interrogate glioma metabolism. A key feature of our approach is the use of an adapted formulation of HPLM,^27^ a medium that approximates the nutrient composition of human plasma. Importantly, we show that this SXO-adapted HPLM formulation supports glioma explant culture while preserving native features of tumor architecture and cell composition. When combined with spatial transcriptomics and isotope tracing, this approach captures metabolic programs that reflect both tumor-intrinsic and microenvironmental activity. Importantly, glioma explants cultured in HPLM exhibit immune and metabolic transcriptional responses not observed in traditional media, suggesting that nutrient context actively shapes the tumor transcriptional state. These responses support the application of HPLM for the discovery of metabolic programs that govern interactions between tumor cells and the TME—features that are lost in neurosphere GSC monocultures.

While GSC models provide valuable insights into tumor-intrinsic metabolic programs, surveying metabolism in glioma explants allows these same programs to be examined in the context of immune and stromal interactions. Our tissue explant model reveals stromal contributions to glioma metabolism that are not evaluable in GSC monocultures. Indeed, metabolites that are regulated by stromal cell metabolism, such as astrocyte-derived GDP-mannose, can be effectively studied using our comparative stable isotope tracing approach. This approach has the advantage of directly measuring both metabolite levels and metabolic activities, rather than relying on expression of metabolic enzymes as a proxy.

The heterogeneity of GBM and underlying transcriptional subtypes represents an important aspect of the biology of these tumors.^50^ Our approach captures metabolic features of GBM transcriptional subtypes, including increased flux through the pyrimidine degradation pathway in mesenchymal glioma cells. Building on prior work in other cancers,^51^ we demonstrate that inducing mesenchymal cell state transitions in GSCs is sufficient to activate pyrimidine degradation. Our study, therefore, deepens our understanding of connections between metabolic plasticity and inflammatory and transcriptional cues within the glioma microenvironment.

By enabling analysis of both cell-autonomous and non-cell-autonomous metabolic programs, our platform enhances the resolution with which glioma metabolism can be interrogated. The ability to maintain intercellular interactions in a nutrient context that is physiologically accurate positions this approach to uncover actionable metabolic dependencies that are tightly coupled to the glioma TME. Stable isotope tracing in HPLM provides a functional platform for evaluating metabolic phenotypes that may not emerge in non-physiologic conditions, opening avenues for therapeutic discovery based on nutrient-sensitive vulnerabilities.

Our study marries advances in tissue culture, stable isotope tracing, and glioma organoid modeling. We acknowledge that this work is subject to several limitations. Although several explant models were used in SXO stable isotope tracing analyses, further validation of our findings in additional models is warranted. We also performed stable isotope tracing at a single timepoint, thereby not allowing for formal evaluation of flux in these models. Tracing with only ^15^ N_2_ glutamine, additionally, may not capture important metabolic differences that could be revealed by tracing other ^13^ C- or ^15^ N-labeled nutrients. Additional studies investigating the metabolism of other metabolic substrates could provide deeper insights into metabolic activities specific to the glioma TME that affect tumor cell viability and growth. Despite these limitations, our findings lay the groundwork for improving the physiologic relevance of in vitro studies of glioma metabolism.

## Supplementary Material

noaf248_Supplementary_Data

## Data Availability

Raw histopathology images and NanoString data will be shared by the lead contact upon request. Metabolomics datasets are available on the NIH National Metabolomics Data Repository (NMDR Accession: ST004507). RNA sequencing of GSC lines was previously published by Wu et al.^49^ butare included along with RNA sequencing analysis of NHA cells for ease of reanalysis in the NCBI Gene Expression Omnibus (GEO Accession: GSE312748). Any additional information required to reanalyze the data reported in this paper is available from the lead contact upon request.
