# Supplementary material for: Stable isotope tracing in human plasma-like medium reveals metabolic and immune modulation of the glioblastoma microenvironment
Source: Neuro Oncol. 2025 Oct 25;28(2):415–29. doi: 10.1093/neuonc/noaf248 (PMC12979037; doi:10.1093/neuonc/noaf248)
Supplement: noaf248_Supplementary_Data [file noaf248_supplementary_data.zip › N-O-D-25-00478R1_-_Supplementary_Materials.docx]

**Supplementary Materials**

**Supplementary Methods**

*Cell Culture (continued)*

BT054 (female, RRID: CVCL_N707) cells were obtained from S. Weiss at the University of Calgary^1^. TS516 (sex unknown, RRID: CVCL_A5HY) and TS603 (sex unknown, RRID: CVCL_A5HW) cells were obtained from I. Mellinghoff at Memorial Sloan-Kettering Cancer Center^2^. HK157 (female), HK211 (female), HK213 (male), HK252 (male), and HK308 (male) cells were obtained from H. Kornblum at the University of California Los Angeles^3^. MGG152 (male) cells were obtained from D. Cahill at Massachusetts General Hospital^4^. HOG (human oligodendroglioma, male, RRID: CVCL_D354) cells were obtained from P. Paez at SUNY University at Buffalo. HEK293T (female, ATCC CRL-3216, RRID: CVCL_0063) cells were obtained commercially. BT054, HK157, TS516, TS603, UTSW5, UTSW63, and UTSW71 cells were cultured in NeuroCult NS-A Basal Medium (Human) with Proliferation Supplement (STEMCELL Technologies 05751), supplemented with EGF (20 ng/mL, GoldBio 1150-04-100), bFGF (20 ng/mL, GoldBio 1140-02-10), heparin (2 µg/mL, STEMCELL Technologies 07980), penicillin/streptomycin (100 U/mL and 100 μg/mL, respectively, Thermo Fisher 15140148), amphotericin B (250 ng/mL, Gemini Bio-Products 400104), and Plasmocin (250 ng/mL, InvivoGen ant-mpp). HK157 (when used for RNA sequencing assays), HK211, HK252, and HK308 cells were cultured in DMEM-F12 medium (Thermo Fisher 11320033) supplemented with glutamine (3mM, Thermo Fisher 25030081), B27 (1×, Thermo Fisher 17504044), EGF (20 ng/mL, GoldBio 1150-04-100), bFGF (20 ng/mL, GoldBio 1140-02-10), heparin (2 µg/mL, STEMCELL Technologies 07980), penicillin/streptomycin (50 U/mL and 50 μg/mL, respectively, Thermo Fisher 15140148), amphotericin B (125 ng/mL, Gemini Bio-Products 400104), and Plasmocin (250 ng/mL, InvivoGen ant-mpp). MGG152 cells were cultured in Neurobasal Medium (Thermo Fisher 21103049) supplemented with glutamine (3mM, Thermo Fisher 25030081), B27 (1×, Thermo Fisher 17504044), N2 (0.25×, Thermo Fisher 17502048), EGF (20 ng/mL, GoldBio 1150-04-100), bFGF (20 ng/mL, GoldBio 1140-02-10), heparin (2 µg/mL, STEMCELL Technologies 07980), penicillin/streptomycin (50 U/mL and 50 μg/mL, respectively, Thermo Fisher 15140148), amphotericin B (125 ng/mL, Gemini Bio-Products 400104), and Plasmocin (0.25 µg/mL, InvivoGen ant-mpp). HOG cells were cultured in IMDM (Thermo Fisher 12440061) supplemented with fetal bovine serum (10%, GeminiBio 100-106) and penicillin/streptomycin (50 U/mL and 50 μg/mL, respectively, Thermo Fisher 15140148). Cells were cultured in 5% CO2 and at ambient oxygen at 37°C. All cells were routinely evaluated for mycoplasma contamination and confirmed to be negative.

*Cloning, Transfection, and Viral Transduction (continued)*

pLV-EF1α-DEST-IRES-Puro was generated from a pLV-EF1α-MCS-IRES-Puro (VB241113-1260bju) plasmid backbone synthesized by VectorBuilder. Gateway destination cassette was amplified by PCR from pLenti-EF1α-DEST-IRES-Neo using the following primers:

MluI-attR1-LEIN_F: TAAGCAACGCGTTGTCGACGAATTCGGATCCG

LEIN-attR2-XbaI_R: TAAGCATCTAGATAATTAAGTTTAAACGCGGCCACCAC

PCR products were gel purified. PCR product and each plasmid backbone were then digested with MluI and XbaI, ligated, and transformed into XL10-Gold Ultracompetent Cells (Agilent 200315). Final products were confirmed by whole plasmid sequencing (Plasmidsaurus). DPYD-3xFLAG (based on NM_000110.4) was synthesized by Twist Biosciences in the Gateway-compatible pTwist-ENTR vector, then introduced to pLV-EF1α-DEST-IRES-Puro by a Gateway LR reaction (Thermo Fisher 11791020). Lentivirus was generated by cotransfection of HEK293T cells with DPYD expression vector, psPAX2 (Addgene 12260, a gift from Didier Trono), and pMD2.G (Addgene 12259, a gift from Didier Trono) at a ratio of 4:3:1 using TransIT-LT1 transfection reagent (Mirus Bio MIR2304). Virus-containing media were collected 48 and 72 hours after transfection, passed through a 0.45 μm filter (Corning 431220), divided into 1 mL aliquots, and frozen at -80°C. HOG cells were plated at a density of 0.15 × 10^6^ cells per well in a 6-well plate. The next day, polybrene (8 µg/mL, MedChemExpress HY-112735) and 1 mL viral supernatant was added. Plates were centrifuged at 4,000 × g for 30 minutes at room temperature and incubated overnight. Cells were then expanded to a 10 cm dish. After initial selection, lines were maintained in 200 ng/mL puromycin.

*Explant SXO Creation and Culture in GOC (continued)*

Tumor tissue was collected from the operating room, directly suspended in ice cold Hibernate A (BrainBits HA), and transferred to the laboratory on ice within 30 minutes of explantation. Tumor pieces were moved into RBC lysis buffer (Thermo Fisher 00433357) and incubated at room temperature for 10 minutes with rocking. Tumor pieces were then washed with Hibernate A containing Glutamax (2 mM, Thermo Fisher 35050061), penicillin/streptomycin (100 U/mL and 100 μg/mL, respectively, Thermo Fisher 15140148), and Amphotericin B (250 ng/mL, Gemini Bio-Products 400104). Tissues were cut into SXOs using a 750 µm^2^ internal diameter needle (SAI Infusion Technologies B18-150) and plated, one per well, in a 24-well ultra-low adherence plate (Corning 3473) in 1 mL Glioma Organoid Complete Medium (GOC)^5^.

*Culture in Glioma Stem-Like Cell (GSC) HPLM (continued)*

GSC HPLM was supplemented with B27 (1×, Thermo Fisher 17504044), N2 (0.25×, Thermo Fisher 17502048), EGF (20 ng/mL, GoldBio 1150-04-100), bFGF (20 ng/mL, GoldBio 1140-02-10), heparin (2 µg/mL, STEMCELL Technologies 07980), penicillin/streptomycin (100 U/mL and 100 μg/mL, respectively, Thermo Fisher 15140148), amphotericin B (250 ng/mL, Gemini Bio-Products 400104), and Plasmocin (250 ng/mL, InvivoGen ant-mpp). For isotopically labeled GSC HPLM, GSC HPLM was prepared as above, substituting equimolar either ^15^N_2_ glutamine (Cambridge Isotope Laboratories NLM-1328) or amide-^15^N glutamine (Cambridge Isotope Laboratories NLM-557) for unlabeled glutamine. Stocks of GSC HPLM were used within a maximum of 1 week after preparation. Prior to experiments conducted in GSC HPLM, GSCs were first cultured for 24 hours in a mixture of 50% NeuroCult and 50% GSC HPLM, then for 24 hours in 100% GSC HPLM.

*Explant Culture in SXO HPLM (continued)*

HPLM was prepared as previously described^6–8^ but without addition of dialyzed fetal bovine serum. HPLM was then supplemented with B27 without Vitamin A (1×, Thermo Fisher 12587010), N2 (1×, Thermo Fisher 17502048), 2-mercaptoethanol (55µM, Thermo Fisher BP176-100), and human insulin (2.375-2.875µg/mL, Millipore Sigma I9278). For isotopically labeled SXO HPLM, SXO HPLM was prepared as above, substituting equimolar ^15^N_2_ glutamine (Cambridge Isotope Laboratories NLM-1328) for unlabeled glutamine. Stocks of SXO HPLM were used within a maximum of 1 week after preparation. Prior to tracing experiments conducted in SXO HPLM, SXOs were first cultured for 24 hours in a mixture of 50% GOC and 50% SXO HPLM, then for 24 or 120 hours in 100% SXO HPLM, replacing HPLM every 24 hours.

*5-fluorouracil Treatment and Flow Cytometry (continued)*

HOG cells expressing either empty vector or DPYD-3xFLAG were plated in 24-well plates at 0.3 × 10^5^ cells per well. 24 hours later, either 300 µM 5-fluorouracil or DMSO was added to wells. 72 hours later, AnnexinV-FITC (BD Biosciences 556547) staining was performed according to the manufacturer’s instructions. DAPI staining was performed at 100 ng/mL final concentration. Flow cytometry data were processed using FCS Express software (De Novo). Dead cells included those that were AnnexinV+/DAPI-, AnnexinV-/DAPI+, or AnnexinV+/DAPI+. Data were normalized to set cell death = 0% in DMSO-treated cells.

*Histology and Immunohistochemistry (continued)*

SXOs were fixed in 10% formalin for 1 hour, washed, and suspended in 70% ethanol. Samples were embedded in paraffin and sectioned at 4μm prior to staining. Histology and immunohistochemistry images were processed using FIJI (1.53f51, imagej.net/software/fiji, RRID:SCR_002285). Nuclei and diaminobenzidine positivity in immunohistochemical stains were quantified with a semi-automated trained object classifier, implemented in QuPath (0.3.1, qupath.github.io, RRID:SCR_018257).

*Immunoblotting (continued)*

Cells were lysed in EBC lysis buffer containing a protease and phosphatase inhibitor cocktail (Thermo Fisher 78440). Primary antibodies used were: DPYD (Proteintech 27662-1-AP, 1:1,000, rabbit polyclonal, RRID: AB_2880941), FLAG (Millipore Sigma F1804, 1:1,000, mouse monoclonal, RRID: AB_262044), and GAPDH (Cell Signaling Technologies 5174, 1:1,000, rabbit monoclonal, RRID: AB_10622025). HRP-conjugated secondary antibody incubation was performed with anti-Mouse IgG (Cell Signaling Technologies 7076, 1:2,000, horse polyclonal, RRID: AB_330924) and anti-Rabbit IgG (Cell Signaling Technologies 7074, 1:2,000, goat polyclonal, RRID: AB_2099233).

*Liquid Chromatography-Mass Spectrometry (LC-MS) (continued)*

For stable isotope tracing experiments, SXOs were washed in ice cold saline prepared in LC-MS grade water (Thermo Fisher W6500), then snap-frozen and stored at -80°C until analysis. Accurate masses were obtained using an analytical balance. 80% LC-MS grade acetonitrile (Thermo Fisher A9554) prepared in LC-MS grade water was added at 100µL per mg of tissue to snap-frozen SXOs on ice, followed by homogenization by manual agitation. Homogenate was vortexed for 20 minutes at 4°C, then centrifuged for 10 minutes at 21,100 × g at 4°C. Supernatant was transferred to a fresh microcentrifuge tube and centrifuged again for 10 minutes at 21,100 × g at 4°C. For GSCs, neurospheres were harvested from 6-well plates, followed by the addition of ice-cold saline prepared in LC-MS grade water. GSCs were transferred to microcentrifuge tubes and centrifuged for 1 minute at 21,100 × g at 4°C. Supernatant was aspirated, then cell pellets were snap-frozen and stored at -80°C. Metabolites were extracted in 80% Acetonitrile at a concentration of 1,000 cells/µL, vortexed for 20 minutes at 4°C, and centrifuged for 10 minutes at 21,100 × g at 4°C. Supernatant was transferred to a fresh microcentrifuge tube and centrifuged again for 10 minutes at 21,100 × g at 4°C. For both SXOs and GSCs, final supernatant was transferred to a glass vial for LC-MS analysis.

After data acquisition, peaks were integrated using El-Maven software (0.12.0, Elucidata). Total ion counts were quantified using TraceFinder software (5.1 SP2, Thermo Fisher). Peaks were normalized to total ion counts using the R statistical programming language. Correction for natural abundance of the ^15^N isotope was accomplished using the R script Accucor^9^. Total fractional enrichment was calculated by subtracting the fractional abundance of the M+0 isotopologue from 1 for each metabolite.

*Digital Spatial Profiling (DSP) Assay (continued)*

After deparaffinization and rehydration, 4μm formalin‐fixed paraffin‐embedded (FFPE) tissue slides were hybridized and incubated at 37°C overnight with morphological immunofluorescent biomarkers and DSP probes from the Cancer Transcriptome Atlas panel (NanoString Technologies). Slides were scanned on the GeoMx DSP instrument to produce a digital image displaying fluorescent visualization markers. Spatially resolved ROIs were selected based on fluorescent markers. DSP probes conjugated to target‐specific activated oligos were collected for each ROI and aliquoted into 96‐well plates. Collection plates were dehydrated at 65°C for 1-2 hours on a thermal cycler with an open top and a breathable AeraSeal film (Excel Scientific A9224). Samples were reconstituted with diethyl pyrocarbonate-treated RNase/DNase-free water, and library preparation was completed with eighteen amplification cycles. Libraries were quantified on an Agilent 4200 TapeStation and pooled for sequencing on an Illumina NextSeq 2000 with a P3 50 flow cell. Whole transcriptome gene sequencing was performed on individual ROI tubes by the University of Pittsburgh Health Sciences Sequencing Core, Rangos Research Center, UPMC Children’s Hospital of Pittsburgh. Resulting FASTQ files were decoded and processed into count files using the NanoString GeoMx NGS Pipeline (2.0.21, NanoString Technologies) in the Illumina BaseSpace Sequencing Hub. Count files were uploaded to the GeoMx DSP instrument and indexed to corresponding slide scans for analysis. Gene counts were mapped to selected ROIs and normalized after a quality check for the following parameters in each ROI: raw read count >1,000, >80% of reads aligned, sequencing saturation >50%, negative probe geometric mean >10 in background, count of nuclei per ROI >200, and surface area per ROI <16,000 µm^2^.

CIBERSORT (RRID:SCR_016955), a deconvolution algorithm built on nine normalized gene expression profiles to characterize cell composition^10^, was used to estimate cell population proportions based on the leukocyte signature matrix 22. CIBERSORT was run for 1,000 permutations, and samples with a CIBERSORT *p* value below 0.05 were included for subsequent analyses. Gene set enrichment analysis (GSEA; 4.2.3; Broad Institute, Inc., Massachusetts Institute of Technology, and Regents of the University of California; RRID:SCR_003199) based on the Molecular Signatures Database (RRID:SCR_016863) was performed to compare SXOs cultured in HPLM to SXOs cultured in GOC. Clinical next-generation sequencing of the parental tumor for SXO210 was performed on FFPE-preserved tissue and paired germline DNA from patient saliva by the UT Southwestern Clinical NGS laboratory (CLIA ID 45D0861764).

*Quantification and Statistical Analysis (continued)*

SXOs were allocated to experiments randomly and samples were processed in an arbitrary order. All statistical tests were two-sided, where applicable. Student’s *t*-test was used to assess the statistical significance of a difference between the two groups. One-way ANOVA was used to assess the statistical significance of differences between three or more groups. Linear regression analysis and Pearson’s correlation coefficient were used to assess correlation between two variables.

**Supplementary Data**

**
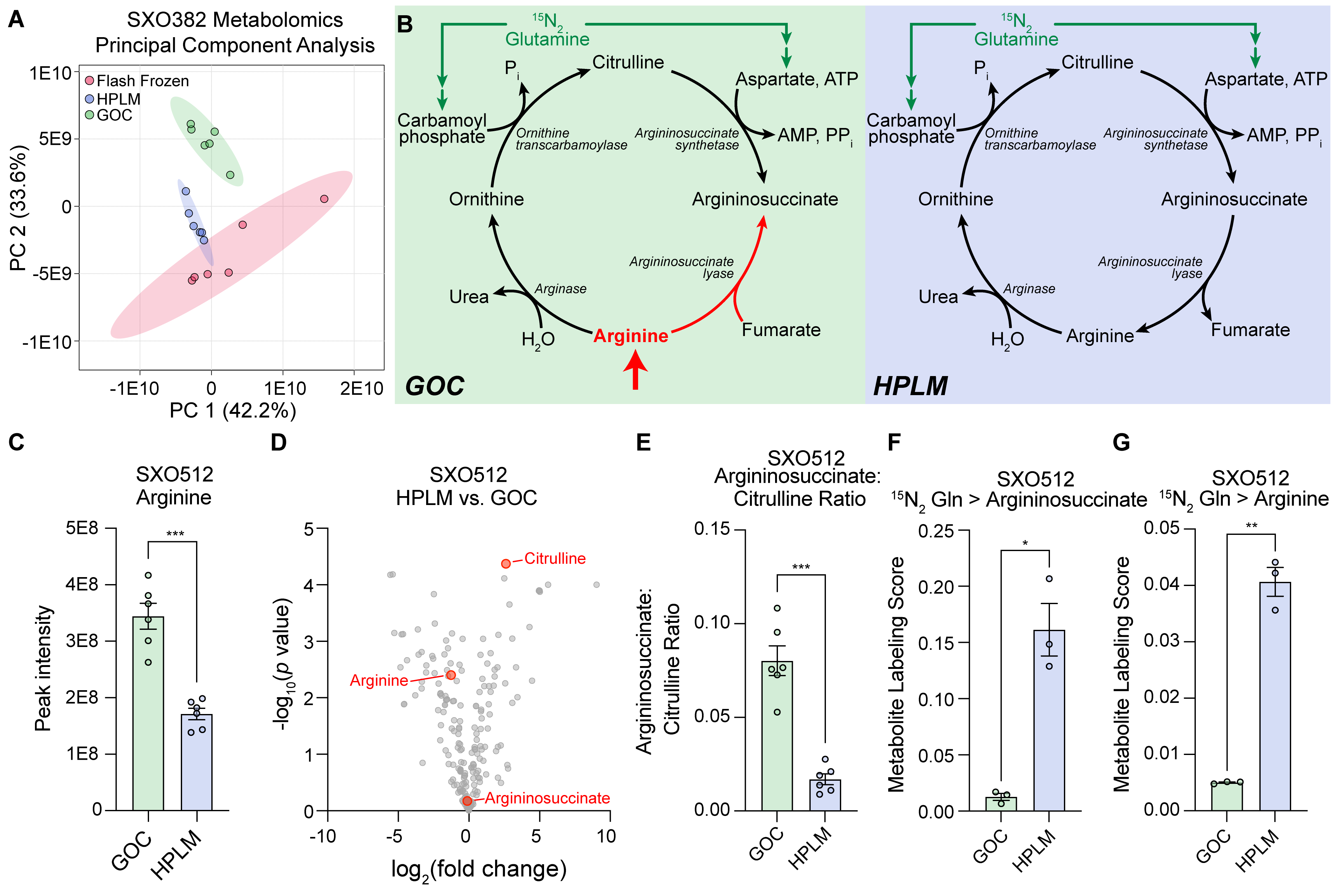
**

**Figure S1. SXO culture in HPLM preserves metabolic fidelity to the parental tumor.** **(A)** Principal component analysis of metabolomics data from SXO382 tissue flash-frozen in the operating room, SXO382 explants cultured in HPLM for 24 hours, and SXO382 explants cultured in GOC for 24 hours. *n* = 6 for each condition. **(B)** Schema of the urea cycle showing entry points of labeled nitrogen from ^15^N_2_ glutamine in GOC and HPLM. Red arrow represents elevated level of arginine in GOC. **(C)** Relative levels of arginine in SXO512 explants cultured in GOC or HPLM. *n* = 6. ****p* < 0.001 (unpaired *t*-test). **(D)** Volcano plot of metabolite levels in SXO512 explants cultured in HPLM versus GOC. Two-tailed *p* values determined by unpaired *t*-test. *n* = 6. **(E)** Argininosuccinate:citrulline ratio in SXO512 explants cultured in GOC or HPLM. *n* =6. ****p* < 0.001 (Welch’s *t*-test). **(F-G)** Stable isotope tracing with ^15^N_2_ glutamine in SXO512 explants cultured in GOC or HPLM. Metabolite labeling scores for **(F)** argininosuccinate or **(G)** arginine are shown. *n* = 3. **p* < 0.05, ***p* < 0.01 (Welch’s *t*-test).


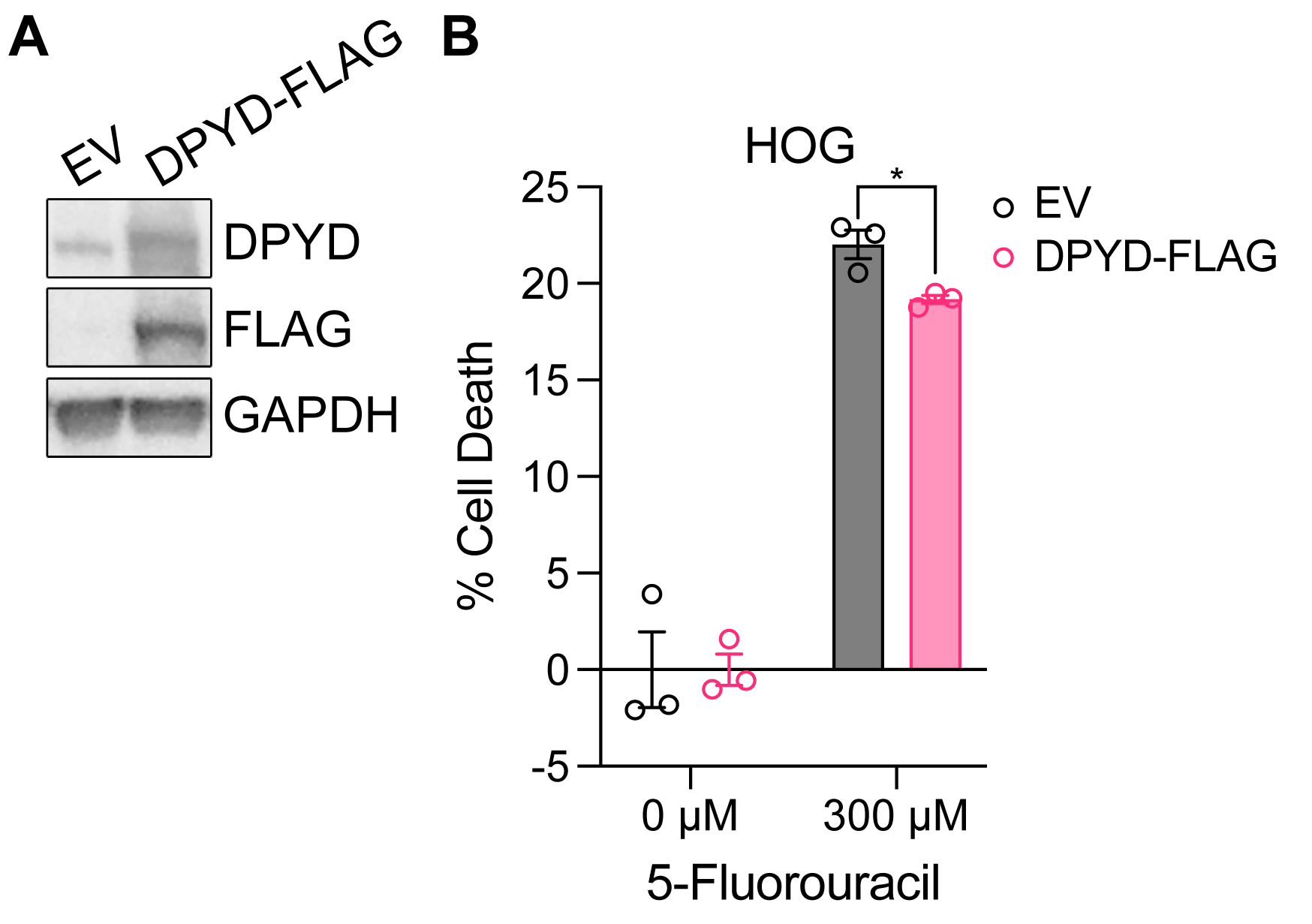


**Figure S2.** **DPYD upregulation decreases sensitivity to 5-fluorouracil. (A)** Immunoblot of DPYD, FLAG, and GAPDH in HOG cells expressing an empty vector (EV) or FLAG-tagged DPYD. *n* = 3. **(B)** Cell death quantification in HOG cells expressing an EV or FLAG-tagged DPYD following treatment with 5-FU or DMSO for 72 hours. *n* = 3. **p* < 0.05 (unpaired *t*-test).

| **Sample ID** | **Model** | **Age** | **Sex** | **Brain Region** | **WHO Grade** | **Diagnosis** | **Primary/**  **Recurrent** |
| --- | --- | --- | --- | --- | --- | --- | --- |
| UTSW63 | Cell Line | 71 | M | Right Temporal | 4 | GBM, IDH-wildtype | Primary |
| SXO210 | SXO | 68 | M | Right Temporal | 4 | GBM, IDH-wildtype | Primary |
| SXO382 | SXO | 60 | F | Left Frontal | 4 | GBM, IDH-wildtype | Recurrent |
| SXO512 | SXO | 68 | F | Right Temporal | 4 | GBM, IDH-wildtype | Recurrent |
| Normal Brain | Primary Tissue | 42 | F | Right Temporal | N/A | Intractable epilepsy | N/A |

**Table S1.** **Clinical characteristics of primary tumor or normal brain specimens.** “Brain Region” corresponds to the location of the tumor or brain specimen at resection. “Diagnosis” corresponds to the results of histopathologic features and molecular studies rendered by a clinical pathologist at resection. Abbreviations: M, male; F, female, GBM, glioblastoma; IDH, isocitrate dehydrogenase; SXO, surgically explanted organoid. IDH status of each primary tumor sample was assessed via immunohistochemistry, next-generation DNA sequencing, or both.

**Table S2 (see Excel file). Gene set enrichment analysis of unsegmented SXOs grown in HPLM versus GOC medium.** Results of GSEA analysis using NanoString-based spatial transcriptomics data collected from SXOs. Gene sets included display positive normalized enrichment score (NES) values in SXOs cultured in HPLM versus GOC medium and relate to one of the following pathways: translation, transcription, metabolism, cell cycle, or immune response.

**Table S3 (see Excel file). Gene set enrichment analysis of immune cell-enriched and immune cell-depleted regions of SXOs grown in HPLM versus GOC medium.** Results of GSEA analysis using NanoString-based spatial transcriptomics data collected from SXOs. Data are binned into CD45^high^ (Tab 1) and CD45^low^ (Tab 2) SXO regions. Reactome terms included were not filtered for relationships with select cellular pathways.

**Table S4 (see Excel file). Comparison of HPLM and GOC medium formulations.** Nutrient concentrations are listed for HPLM medium and GOC medium. Percent differences in the concentrations of each nutrient in the two medium types are displayed in Column D.**Supplementary References**

1. Kelly JJ, Blough MD, Stechishin OD, et al. Oligodendroglioma cell lines containing t(1;19)(q10;p10). *Neuro Oncol*. 2010;12(7):745-755. doi:10.1093/neuonc/noq031

2. Rohle D, Popovici-Muller J, Palaskas N, et al. An Inhibitor of Mutant IDH1 Delays Growth and Promotes Differentiation of Glioma Cells. *Science*. 2013;340(6132):626-630. doi:10.1126/science.1236062

3. Laks DR, Ta L, Crisman TJ, et al. Inhibition of Nucleotide Synthesis Targets Brain Tumor Stem Cells in a Subset of Glioblastoma. *Molecular Cancer Therapeutics*. 2016;15(6):1271-1278. doi:10.1158/1535-7163.MCT-15-0982

4. Wakimoto H, Tanaka S, Curry WT, et al. Targetable Signaling Pathway Mutations Are Associated with Malignant Phenotype in IDH-Mutant Gliomas. *Clinical Cancer Research*. 2014;20(11):2898-2909. doi:10.1158/1078-0432.CCR-13-3052

5. Jacob F, Salinas RD, Zhang DY, et al. A Patient-Derived Glioblastoma Organoid Model and Biobank Recapitulates Inter- and Intra-tumoral Heterogeneity. *Cell*. 2020;180(1):188-204 e22. doi:10.1016/j.cell.2019.11.036

6. Cantor JR, Abu-Remaileh M, Kanarek N, et al. Physiologic Medium Rewires Cellular Metabolism and Reveals Uric Acid as an Endogenous Inhibitor of UMP Synthase. *Cell*. 2017;169(2):258-272 e17. doi:10.1016/j.cell.2017.03.023

7. Leney-Greene MA, Boddapati AK, Su HC, Cantor JR, Lenardo MJ. Human Plasma-like Medium Improves T Lymphocyte Activation. *iScience*. 2020;23(1):100759. doi:10.1016/j.isci.2019.100759

8. Rossiter NJ, Huggler KS, Adelmann CH, et al. CRISPR screens in physiologic medium reveal conditionally essential genes in human cells. *Cell Metabolism*. 2021;33(6):1248-1263.e9. doi:10.1016/j.cmet.2021.02.005

9. Su X, Lu W, Rabinowitz JD. Metabolite Spectral Accuracy on Orbitraps. *Anal Chem*. 2017;89(11):5940-5948. doi:10.1021/acs.analchem.7b00396

10. Newman AM, Steen CB, Liu CL, et al. Determining cell type abundance and expression from bulk tissues with digital cytometry. *Nat Biotechnol*. 2019;37(7):773-782. doi:10.1038/s41587-019-0114-2
